# Effect of endotracheal tube cuff pressure on postoperative outcomes in Laparoscopic cholecystectomy

**DOI:** 10.6026/973206300220146

**Published:** 2026-01-31

**Authors:** Mukesh Kumar Prasad, Singh N.K, Singh V.K

**Affiliations:** 1 Department of Anesthesia, TMMC and RC, Teerthanker Mahveer University, Moradabad, Uttar Pradesh, India; 2 Department of Surgery, TMMC & RC, Teerthanker Mahveer University, Moradabad, Uttar Pradesh, India; 3 Department of Medicine, TMMC and RC, Teerthanker Mahveer University, Moradabad, Uttar Pradesh, India

**Keywords:** Endotracheal tube cuff pressure, Nitrous oxide, Hemodynamic changes, Postoperative complication, Laparoscopic cholecystectomy

## Abstract

Despite widespread use of nitrous oxide in laparoscopic cholecystectomy, its impact on endotracheal tube cuff pressure, hemodynamics and postoperative complications remains insufficiently characterized. This study compared the effects of air and nitrous oxide as inflating agents on haemodynamic parameters, postoperative complications and endotracheal tube cuff pressure in patients undergoing laparoscopic cholecystectomy. A total of 148 patients were monitored for intraoperative vital signs and cuff pressures at 30, 60 and 90 minutes. Postoperative issues such as dysphagia, hoarseness and sore throat were assessed immediately and 24 hours after surgery. Nitrous oxide significantly increased cuff pressure (p < 0.001) and was associated with a higher incidence of sore throat (25.68% vs. 9.46%, p = 0.007). These findings highlight the importance of careful cuff pressure monitoring to reduce postoperative complications and ensure patient safety.

## Background:

Laparoscopic cholecystecto is a widely used minimally invasive surgical procedure for the treatment of gallbladder diseases such as cholelithiasis and cholecystitis [1]. It offers substantial advantages over open surgery, such as a faster recovery and fewer complications; however, it also has potential hazards and unanticipated consequences [2]. The use of endotracheal tubes and airway management has a significant impact on intraoperative and postoperative outcomes. The possible effects of pressure within the endotracheal tube closure on patient outcomes during and after surgery have sparked considerable interest [3]. The cuff of the endotracheal tube is critical for generating an airtight seal in the trachea, preventing aspiration and ensuring successful ventilation. Inadequate management of sleeve pressure, whether due to overinflation or underinflation, can cause a variety of complications [4]. Inadequate inflation increases the risk of microaspiration and other pulmonary complications, whereas excessive cuff pressure can injure the tracheal mucosa [5]. Nitrous oxide, when used as an anesthetic, can enter the cuff during procedures, causing a gradual rise in pressure [6]. Laparoscopic cholecystectomy presents unique challenges, particularly in the establishment of a viable operative field within the abdominal cavity. The insufflation of carbon dioxide into the abdominal cavity is a critical component of the pneumo peritoneum procedure [7]. The increased intra- abdominal pressure from the pneumo peritoneum may cause significant changes in blood flow, which could affect the stability of the cuff pressure and the management of the airway [8]. In order to ensure patient safety and improve outcomes, it is necessary to monitor and modify cuff pressure in conjunction with the effects of anesthetic gases and the changes above [9]. Laparoscopic procedures frequently employ nitrous oxide due to its rapid onset and recovery characteristics. Nevertheless, it is imperative to continuously monitor the cuff pressure, as it has a propensity to infiltrate the endotracheal tube cuff [10]. As an alternative inflation medium, air may be a safer choice for sustaining consistent pressure, as it enhances the stability of the cuff pressure in comparison to nitrous oxide [11]. By investigating the distinct impacts of nitrous gas and air on cuff pressure, it is possible to improve airway control during laparoscopic interventions. Postoperative complications, such as hoarseness, sore larynx, tracheal stenosis, or nerve injury, may result from inadequate cuff pressure, thereby exacerbating patient suffering and healthcare expenditures [12]. Additionally, elevated cuff pressures may more effectively manage the hemodynamic abnormalities, such as fluctuations in blood pressure, heart rate and systemic vascular resistance that arise during laparoscopic cholecystectomy [13]. It is essential to comprehend these relationships in order to develop anesthetic procedures that ensure airway safety while maintaining hemodynamic stability. Therefore, it is of interest to investigate the effects of nitrous oxide and air on the pressure of the endotracheal tube cuff during laparoscopic cholecystectomy, with a particular emphasis on their impact on postoperative complications and hemodynamic parameters.

## Methodology:

This prospective comparison study was conducted in June 2024 in the Department of Anaesthesiology at Teerthankar Mahaveer Medical College & Research Centre in Moradabad, with the agreement of the College Research Committee and the Ethical Committees. Before their involvement, all patients received detailed information about the surgery, including potential side effects like hypotension and bradycardia. The study required them to provide informed consent.

## Sample size calculation:

The study used a convenience sampling method to determine the required sample size with a standard formula for sample size computation. The study's criteria required a minimum sample size of 74 patients per group, with a 95% confidence interval, 80% power, a standard deviation of 11.5 and a mean difference of 5.3. Participants were randomly assigned to two groups using the Chit and Box method. Group A received isoflurane (1-2%) in a nitrous oxide/oxygen combination, while Group B received it in an oxygen/air mixture.

## Inclusion and exclusion criteria:

Adults aged 18 to 65 who underwent laparoscopic cholecystectomy, had an ASA physical status of I or II and were provided written informed permission were considered eligible. The study excluded patients who had more than two intubation attempts, had a nasogastric tube, oral airway, pharyngeal pack, or endotracheal tube lubricant, or were actively suffering from an upper respiratory tract infection.

## Data collection procedure:

All participants were instructed to refrain from oral intake starting at midnight following a thorough pre-anesthesia evaluation. Premedication included intravenous administration of midazolam (1 mg), glycopyrrolate (200 mcg), dexamethasone (8mg) and fentanyl (50 mcg). Baseline vital signs were recorded in the operating room using standard monitoring equipment, including electrocardiography, non- invasive blood pressure monitoring and pulse oximetry. After three minutes of preoxygenation, intravenous propofol (2-2.5 mg/kg) was administered to induce anesthesia. Neuromuscular blockade was achieved with intravenous vecuronium (0.1mg/kg) and intubation was performed by a single experienced anesthesiologist using appropriately sized endotracheal tubes (7 mm for females and 8 mm for males). The endotracheal tube cuff was inflated with air and its pressure was carefully monitored with an aneroid manometer to maintain it at 25 cm H_2_O during the surgery. Essential modifications were executed with a syringe to sustain the desired pressure. Anesthesia was maintained by isoflurane (1-2%) in conjunction with either an oxygen/air combination or a nitrous oxide/oxygen mixture, depending on the designated group. Fluctuations in cuff pressure were noted, particularly during stages of increased intra-abdominal pressure induced by carboperitoneum or positional alterations, such as the Trendelenburg position. We administered further vecuronium bromide dosages (0.02 mg/kg) as needed to ensure adequate muscle relaxation. After the procedure was finished, the isoflurane and associated gas mixture were stopped and the oropharynx was carefully suctioned before extubation. Postoperatively, patients were observed in the surgical ward for six hours and evaluated for complications such as laryngeal pain, hoarseness, dysphagia, oxygen desaturation, vomiting and post- obstructive pulmonary edema.

## Statistical analysis:

The data was collected and analyzed thoroughly using SPSS software (version 25.0, SPSS Inc., Chicago, USA). Descriptive statistics, such as standard deviations and means, were used to summarize continuous data. The t-test and chi-square test, were used to compare the two groups. Pearson correlation analysis was used to evaluate the association between cuff pressure and haemodynamic parameters, as well as the correlations between systolic and diastolic pressures and heart rate, using a variety of time intervals. A p-value less than 0.05 indicate statistical significance.

## Results:

The demographic profile of participants in Groups A and B is compared in Table 1. The gender distribution was identical in both categories; with males comprising 41.89% of Group A and 45.95% of Group B. Group A was composed of 58.11% females. In comparison, Group B was composed of 54.05% females. There was no statistically significant difference in the gender distribution (p-value = 0.79). The mean age of Group A members was 39.30 ± 10.48 years, which was comparable to the mean age of Group B members, 41.52 ± 9.56 years. The p-value of 0.77 suggested that there was no statistically significant difference in the ages of the participants. The ASA physical status classification classified the majority of individuals in both categories as Grade I, with 68.92% in Group A and 64.87% in Group B. Group A and Group B designated a lesser percentage as Grade II, with 31.08% and 35.13%, respectively. There were no statistically significant differences in the distribution of ASA grades between the two groups (p = 0.88). Table 2 provides a thorough comparison of vital signs, including systolic and diastolic blood pressure, mean arterial pressure (MAP), heart rate, oxygen saturation (SpO_2_) and cuff pressure, between Groups A and B at three distinct time intervals: 30 minutes, 1 hour and 1.5 hours. Group A exhibited a significantly higher systolic blood pressure at 30 minutes (133.86 ± 8.62 vs. 125.34 ± 13.03; p = 0.00) and 1 hour (120.7 ± 12.03 vs. 112 ± 16.86; p = 0.00). However, the disparity decreased by 1.5 hours (p = 0.71). Diastolic blood pressure did not vary considerably at the outset; however, it was significantly higher in Group A at 1.5 hours (72.31 ± 8.49 vs. 68.4 ± 9.5; p = 0.0002). The mean arterial pressure (MAP) and heart rate were consistent at all intervals and no statistically significant differences were observed. SpO_2_ was marginally elevated in Group A at 30 minutes (99 ± 0.56 vs. 98.8 ± 0.68; p = 0.006); however, no subsequent alterations were observed. The cuff pressure in Group A was consistently and substantially elevated at all intervals, with p-values of 0.00. These data emphasize significant disparities in specific metrics, such as cuff pressure and blood pressure, while other vital indicators remained consistent and comparable across the groups over time. Systolic blood pressure did not exhibit any significant relationships in either group at any interval. Table 3 shows the Pearson correlation of hemodynamic parameters among both groups. Diastolic blood pressure in Group B demonstrated a substantial positive correlation after one hour (r = 0.3, p = 0.009) despite the absence of significant alterations at other intervals. In both groups, the mean arterial pressure consistently exhibited no correlation with time.

**Figure 1:**
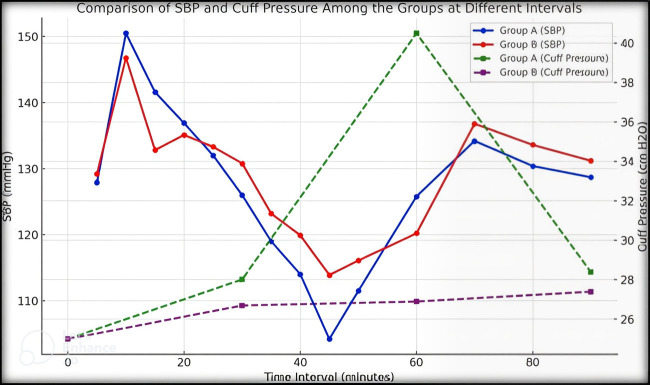
Comparison of SBP and cuff pressure among the groups at different intervals

**Figure 2:**
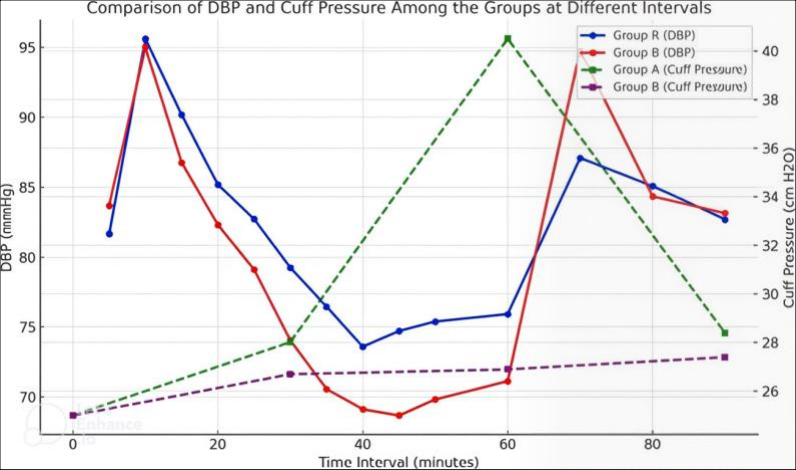
Comparison of DBP and Cuff pressure among the groups at different intervals

**Figure 3:**
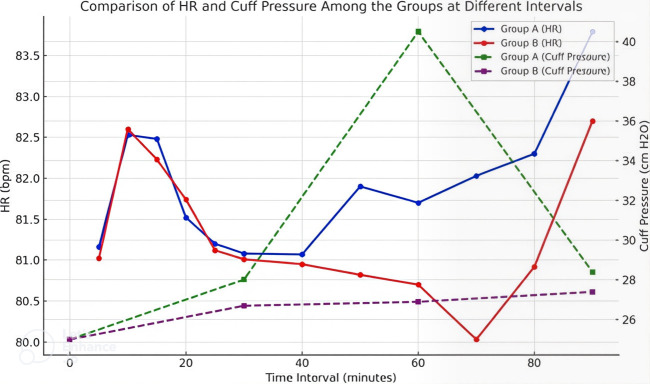
Comparison of HR and cuff pressure among the groups at different intervals

**Figure 4:**
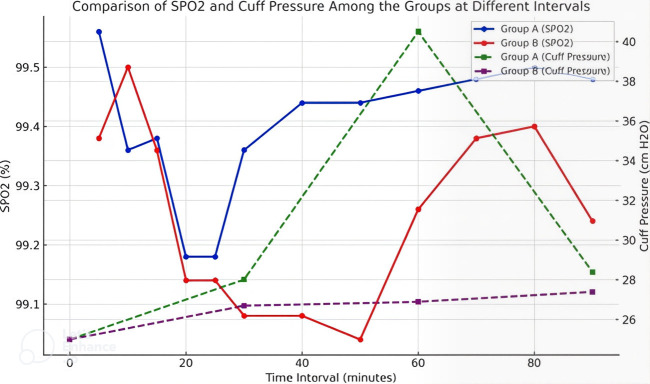
Comparison of SPO_2_ and cuff pressure among the groups at different intervals

Group A (r = -0.2, p = 0.01) and Group B (r = -0.3, p = 0.002) both exhibited significant negative correlations with heart rate at 30 minutes. However, these correlations decreased in successive intervals. The oxygen saturation (SpO_2_) of Group A was positively correlated after 30 minutes (r = 0.24, p =0.03) and significantly negative at 1.5 hours (r = -0.34, p = 0.002) similar to Group B(r = - 0.36, p = 0.001). These results emphasize the temporal and dynamic nature of these physiological variables, with heart rate and SpO_2_ exhibiting particularly significant fluctuations over time. Table 4 examines the correlation between the type of gas used (nitrous oxide or air) and cuff pressure over three time intervals. At 30 minutes, the cuff pressure showed a 2624 modest yet significant positive correlation with nitrous oxide (r = 0.22, p = 0.04), while no significant correlation was observed with air (r = -0.06, p = 0.5). A significant relationship was observed over the 60 minutes, as evidenced by the robust positive correlation observed for both nitrous oxide (r = 0.96, p < 0.001) and air (r =0.91, p < 0.001). The correlations for both gases were not statistically significant and had diminished by 90 minutes (nitrous oxide: r = 0.10, p = 0.37; air: r = 0.018, p =0.87). The results suggest that the correlation between cuff pressure and gas type is most apparent at 60 minutes, while the relationships at earlier and later time intervals are negligible or nonexistent. Depict a comparison of systolic blood pressure and cuff pressure between Groups A and B at different time points. The SBP in both groups shows a distinct pattern with Group A continuously having somewhat higher values than Group B. Group A shows a significant increase in cuff pressure after 30 minutes, peaking at around 60 minutes. The cuff pressure follows a particular trend. In comparison, Group B maintains stable and lower cuff pressure levels throughout the procedure. This image depicts the temporal variations and differences in physiological responses between the two groups. Figure 1 Compares the SBP and cuff pressure among the groups at different intervals. Figure 2 compares the diastolic and cuff pressures of Groups A and B across different time intervals. The diastolic pressures for both groups change dynamically over time with Group A consistently showing lower values than Group B. In terms of cuff pressure, Group A demonstrates a notable rise, reaching its peak at 60 minutes. In contrast, Group B maintains a steady and significantly lower cuff pressure throughout the same period. The figure effectively captures the differences in physiological responses between the groups, highlighting time-dependent variations in cuff pressure. Figure 3 illustrates a comparison of heart rate (HR) and cuff pressure between Groups A and B over various time intervals. Both groups exhibit relatively stable HR trends, with minor fluctuations and overlapping patterns, indicating minimal differences between the groups. In contrast, cuff pressure demonstrates a marked divergence, with Group A showing a steep increase that peaks at 60 minutes, followed by a decline, while Group B maintains a consistently lower and stable cuff pressure throughout. This visualization highlights the significant contrast in cuff pressure dynamics while emphasizing the stability of heart rate across both groups. Figure 4 presents a comparative analysis of SPO_2_ levels and cuff pressure between groups (Group A and Group B) at different time intervals. The left y-axis indicates SPO_2_ levels (%), whereas the right y-axis represents cuff pressure (cm H2O). The SPO_2_ levels of Group A, represented by the blue line, exhibit significant fluctuations, reaching a minimum at approximately 20 minutes, followed by a gradual stabilisation. Conversely, Group B's SPO_2_ (red line) shows a comparable trend, albeit consistently at lower levels. The cuff pressure for Group A, indicated by the green dashed line, exhibits a notable peak at 60 minutes. In contrast, Group B's cuff pressure, represented by the purple dashed line, maintains stability across the intervals. Significant variations in physiological responses are observed among the groups. Table 5 compares the incidence of postoperative complications, including hoarseness, sore throat and dysphagia, between Groups A and B at two intervals: immediately post- anaesthesia care unit and 24 hours postoperatively. Hoarseness was observed in14.86% of Group A and 10.81% of Group B in the PACU, with no significant difference (p = 0.37) and only one case persisted in Group A after 24 hours. Sore throat occurred significantly more frequently in Group A than in Group B in the PACU (25.68% vs. 9.46%; p = 0.007), though it subsided in both groups within 24 hours. Dysphagia was slightly more prevalent in Group A (12.16%) compared to Group B (5.41%) in the PACU, but this difference was not statistically significant (p= 0.24) and no cases were reported after 24 hours. These findings highlight a notable disparity in sore throat incidence between the groups immediately postoperatively, while other complications showed no significant differences and resolved within 24 hours.

## Discussion:

This study investigated the effects of nitrous oxide and air as inflating agents on endotracheal tube cuff pressure, patient hemodynamics and postoperative complications following laparoscopic cholecystectomy. The findings highlight significant differences between the two groups in cuff pressures and hemodynamic parameters, emphasizing the need to consider critical aspects of airway management during therapeutic procedures. The results revealed a significant increase in cuff pressures in the nitrous oxide group compared to the air group at all measured time points. This increase is attributed to the diffusion of nitrous oxide into the cuff, causing a steady rise in pressure. These findings align with previous research which demonstrated that nitrous oxide significantly increases cuff pressure, often exceeding safe limits [14]. The strong positive correlation between nitrous oxide and cuff pressure at 60 minutes underscores the necessity of regular intraoperative monitoring to prevent complications, such as tracheal mucosal injury due to excessive pressure. In terms of hemodynamics, the nitrous oxide group exhibited a significant increase in systolic blood pressure at 30 and 60 minutes after the intervention, supporting the hypothesis that elevated cuff pressure can induce transient hemodynamic changes, particularly during laparoscopic procedures involving pneumoperitoneum. Furthermore, fluctuations in diastolic blood pressure at later time points suggest a delayed response to prolonged nitrous oxide diffusion. Another study reported similar findings, observing hemodynamic variability in patients subjected to various cuff inflation media [15].

Minimal variations in oxygen saturation were observed between the groups and these were not clinically significant. While differences in heart rate were also insignificant, the nitrous oxide group showed a negative association at 30 minutes, indicating a possible autonomic response to increased cuff pressure. This is consistent with findings of a previous study which reported stable oxygenation and negligible heart rate changes under anesthesia, irrespective of the cuff medium used [16]. Postoperative complications were more prevalent in the nitrous oxide group, particularly the incidence of sore throat in the post- anesthesia care unit (25.68% vs. 9.46%). This significant disparity underscores the impact of increased cuff pressure on postoperative discomfort and pain. Although hoarseness and dysphagia were more frequent in the nitrous oxide group, these issues were not statistically significant and resolved within 24 hours. These results align with existing literature, which links higher cuff pressures to increased postoperative throat irritation and discomfort [14, 15- 16]. Given these findings, meticulous intraoperative monitoring of endotracheal tube cuff pressures is essential, mainly when using nitrous oxide. Maintaining pressures within the recommended range of 20-30 cm H2O can mitigate risks such as mucosal damage and postoperative complications. Routine use of manometers and adherence to established cuff pressure monitoring protocols can significantly enhance patient safety and postoperative comfort.

## Limitations:

The study has several limitations that warrant consideration. Although the sample size was adequate to detect significant differences, it may limit the generalizability of the findings to a broader population. The study focused exclusively on patients undergoing laparoscopic cholecystectomy, making it challenging to extrapolate the results to other surgical procedures and settings. Furthermore, the study did not account for patient-specific characteristics, such as body mass index, pre-existing respiratory conditions, or the duration of anesthesia, all of which could influence outcomes.

## Conclusion:

Nitrous oxide as an inflating agent significantly increases endotracheal tube cuff pressure, causing more postoperative sore throat and hemodynamic changes compared to air. These findings highlight the need for careful cuff pressure monitoring to ensure patient safety and reduce complications. Further large-scale studies with longer follow-up are required to validate and enhance intraoperative airway management strategies.

## Figures and Tables

**Table 1:** Demographic presentation of both groups

**Variables**	**Group A**	**Group B**	**p-value**
Gender			0.79
Male	31 (41.89%)	34 (45.95%)	
Female	43 (58.11%)	40 (54.05%)	
Age in years	39.30 ± 10.48	41.52 ± 9.56	0.77
ASA grade			0.88
Grade I	51 (68.92%)	48 (64.87%)	
Grade II	23 (31.08%)	26 (35.13%)	

**Table 2:** Comparison of blood pressure, heart rate and other vital signs between group A and group B over time

	**After 30 minutes**		**After 1 hour**		**After 1.5 hour**		**After 30 minutes**		**After 1 hour**		**After 1.5 hour**	
	**Group A**	**Group B**	**Group A**	**Group B**	**Group A**	**Group B**	**t-test**	**p-value**	**t-test**	**p-value**	**t-test**	**p-value**
Systolic blood pressure	133.86 ± 8.62	125.34 ± 13.03	120.7 ± 12.03	112 ± 16.86	111.64 ± 15.27	112.37 ± 18.72	6.63	0	5.11	0	-0.4	0.71
Diastolic blood pressure	75.1 ± 9.3	74.6 ± 9.3	75.65 ± 11.78	73.6 ± 11.9	72.31 ± 8.49	68.4 ± 9.5	0.46	0.64	1.49	0.14	3.73	0.0002
Mean arterial pressure	80.98 ± 19.28	81.7 ± 15	71.47 ± 21.14	71.7 ± 17.5	76.1 ± 13.1	76.6 ± 13.3	-0.36	0.72	-0.1	0.92	-0.3	0.74
Heart Rate	75.4 ± 12.9	74.16 ± 11.3	76 ± 14.2	76.91 ± 13.1	74 ± 11	72.4 ± 10.3	0.88	0.38	-0.57	0.57	1.29	0.2
SPO_2_	99 ± 0.56	98.8 ± 0.68	98.84 ± 0.68	98.8 ± 0.67	98.87 ± 0.57	98.8 ± 0.57	2.76	0.006	0.51	0.61	1.06	0.29
Cutt pressure	28.14 ± 1.3	26.5 ± 1	40.14 ± 2.43	27.12 ± 1.3	28.12 ± 1.3	27.44 ± 1.09	12.2	0	57.5	0	4.88	0

**Table 3:** Pearson correlation of hemodynamic parameters among both groups

**Variables**	**Group A**		**Group B**	
	**r**	**p-value**	**r**	**p-value**
**Systolic blood pressure**				
After 30 minutes	0.04	0.67	-0.01	0.9
After 1 hour	0.08	0.45	0	0.98
After 1.5 hour	0.09	0.43	-0.02	0.83
**Diastolic blood pressure**				
After 30 minutes	-0.1	0.22	-0.07	0.5
After 1 hour	0.11	0.33	0.3	0.009
After 1.5 hour	0.2	0.07	0.02	0.82
**Mean arterial pressure**				
After 30 minutes	0	0.7	-0.05	0.6
After 1 hour	0.07	0.5	0.04	0.7
After 1.5 hour	0.08	0.45	0.08	0.45
**Heart rate**				
After 30 minutes	-0.2	0.01	-0.3	0.002
After 1 hour	0	0.77	-0.07	0.5
After 1.5 hour	0.06	0.5	0.23	0.04
**SPO_2_**				
After 30 minutes	0.24	0.03	0.24	0.06
After 1 hour	-0.2	0.08	-0.19	0.08
After 1.5 hour	-0.3	0.002	-0.36	0.001

**Table 4:** Correlation analysis of cut-off pressure with nitrous oxide and air at different time intervals

**Cutt off pressure**	**Nitrous Oxide**		**Air**	
	**r**	**p-value**	**r**	**p-value**
After 30 minutes	0.2	0.04	-0.06	0.5
After 60 minutes	1	<0.001	0.91	<0.001
After 90 minutes	0.1	0.37	0.02	0.87

**Table 5:** Comparison of postoperative complications among the groups at different intervals

**Complications**	**Group A**		**Group B**		**p value**
	**N**	**%**	**N**	**%**	
Hoarseness					
PACU	11	14.9	8	10.8	0.37
After 24 hrs	1	1.35	0	0	
Sore Throat					
PACU	19	25.7	7	9.46	0.007*
After 24 hrs	1	1.35	0	0	
Dysphagia					
PACU	9	12.2	4	5.41	0.24
After 24 hrs	0	0	0	0	
